# Antigen binding allosterically promotes Fc receptor recognition

**DOI:** 10.1080/19420862.2018.1522178

**Published:** 2018-10-05

**Authors:** Jun Zhao, Ruth Nussinov, Buyong Ma

**Affiliations:** a Cancer and Inflammation Program, National Cancer Institute, Frederick, Maryland, USA; b Basic Science Program, Leidos Biomedical Research, Inc., Cancer and Inflammation Program, National Cancer Institute, Frederick, Maryland, USA; c Sackler Inst. of Molecular Medicine, Department of Human Genetics and Molecular Medicine, Sackler School of Medicine, Tel Aviv University, Tel Aviv, Israel

**Keywords:** antibody, allostery, conformational selection, allosteric effects

## Abstract

A key question in immunology is whether antigen recognition and Fc receptor (FcR) binding are allosterically linked. This question is also relevant for therapeutic antibody design. Antibody Fab and Fc domains are connected by flexible unstructured hinge region. Fc chains have conserved glycosylation sites at Asn297, with each conjugated to a core heptasaccharide and forming biantennary Fc glycan. The glycans modulate the Fc conformations and functions. It is well known that the antibody Fab and Fc domains and glycan affect antibody activity, but whether these elements act independently or synergistically is still uncertain. We simulated four antibody complexes: free antibody, antigen-bound antibody, FcR-bound antibody, and an antigen-antibody-FcR complex. We found that, in the antibody’s “T/Y” conformation, the glycans, and the Fc domain all respond to antigen binding, with the antibody population shifting to two dominant clusters, both with the Fc-receptor binding site open. The simulations reveal that the Fc-glycan-receptor complexes also segregate into two conformational clusters, one corresponding to the antigen-free antibody-FcR baseline binding, and the other with an antigen-enhanced antibody-FcR interaction. Our study confirmed allosteric communications in antibody-antigen recognition and following FcR activation. Even though we observed allosteric communications through the IgG domains, the most important mechanism that we observed is the communication via population shift, stimulated by antigen binding and propagating to influence FcR recognition.

## Introduction

Immunoglobulin G (IgG) molecules bind to their cognate antigens. The resulting complexes interact either with type I or type II Fc receptors (FcRs) on effector cells and on B cells, modulating both humoral and innate immune processes.^1^ IgG contains four polypeptide chains, two light chains (LC) and two heavy chains (HC). These four chains fold into three domains, two Fab domains that bind antigen and one Fc domain that binds Fc receptors (FcRs).^2^ The Fab domains contain variable and constant domains. The variable domains, especially complementarity-determining regions (CDRs), are mainly responsible for specificity and affinity,^3^ while the constant domains modulate isotype/effector functions.^4^ The Fc domain contains CH2 and CH3 domains. The CH2 domain mainly interacts with FcRs, which are on the cell surface and play pivotal roles in humoral and cellular protection. The Fab and Fc domains are connected by a flexible unstructured hinge region. Fc chains have conserved glycosylation sites at Asn297. Each is conjugated to a core heptasaccharide. They form a biantennary Fc glycan. Thus, three structural elements (Fab, Fc, and glycan) synergistically determine antibody activity.

Antibody-antigen recognition is a complex event that involves antibody conformational transitions mediated by its inherent flexibility.^5–7^ Recent studies suggested allosteric effects during antibody-antigen recognition ^8^, with both the variable and constant domains playing a role.^9–14^ For instance, our recent work on crenezumab suggested that antibodies with identical variable domains, but different constant domains, have significantly different affinities to amyloid beta (Aβ).^15^ Engineering CH and CL in trastuzumab and pertuzumab recombinant models also affect antigen-binding.^16^ A previous study based on over 100 crystal structures of antibody Fab domains in either unbound or bound form indicated a common behavior, with distant CH1-1 loops undergoing significant fluctuations upon antigen binding.^17^

IgGs are the most common template for antibody drugs. Antibody Fc-FcRs interactions are crucial in the design of therapeutic agents, as well as vaccines.^18,19^ One of the most important antibody activities involves killing target cells by triggering antibody-dependent cell-mediated cytotoxicity (ADCC). Fc-optimized antibodies can have higher binding affinity with FcRs and achieve a higher ADCC potency.^20–24^ For example, antibody Fc engineering promotes serial killing mediated by natural killer cells.^21^ Fc-optimized anti-CD25 ^22^ and anti-CD133^24^ antibodies were reported to achieve certain success. Antigen presentation is also an important immune-response step. FcγRs efficiently internalize antigen-antibody (Ag-Ab) complexes, inducing processing of antigens into peptides presented by major histocompatibility complex (MHC) class II molecules. The recognition of p-MHC (peptide-MHC) complexes by T-cell receptors (TCR) triggers further immune reactions.

Fc glycans modulate Fc conformations and functions.^25,26^ While glycan truncation may affect antibody stability,^27^ defucosylation may enhance effector functions.^28^ N-Glycan optimization can also be used to maximize ADCC.^29^ Most intriguing, glycan may also regulate antigen recognition, and it has been reported that core fucosylation of IgG B cell receptor is required for antigen recognition and antibody production.^30^

Antibody effectors can be antigen specific,^31^ indicating the intrinsic connection between antigen recognition and Fc receptor binding. However, the signals that dictate antigen binding, Fc conformational change, and IgG effector function during immune response development remain poorly understood. Whether intramolecular signaling occurs is still debated.^14,32,33^ While the associative hypothesis is attractive, since Fc receptor crosslinking could increase the affinity of antigen–antibody complexes, there is sufficient evidence to support the allosteric hypothesis.^14,32,33^ Elucidation of the allosteric hypothesis is important for understanding the mechanism of recognition, but, by shifting the focus from solely the variable region to the entire antibody molecule, it is also critical for antibody engineering.

Here, we investigate whether antigen binding induces conformational change in the Fc domain and hinge region, and whether an antigen-bound antibody populates conformations that facilitate or inhibit the binding of FcRs to Fc. We selected an Aγ peptide as antigen to minimize antigen size effects, since a larger antigen may introduce uncertainties in sampling antibody states. We focus on human FcγR I (hFcγRI), a major immune receptor expressed in immune cells, macrophages, neutrophils, and dendritic cells. hFcγRI binds IgG1, 3, and 4 with high affinity. hFcγRI contains three subunits, D1, D2, and D3. Structures of the unbound hFcγRI and hFcγRI-IgG1 complex show an asymmetric binding surface, as well as significant conformational change of both hFcγRI D3 and CH2(A) domains.

Our molecular dynamics (MD) simulations showed that Aβ binding leads to large Fab re-orientation into two dominant conformational clusters, as well as open conformations of Fc CH2 domains. The conformations share properties with the dominant states of Aβ-solanezumab-hFcγRI complex. This suggested that Aβ binding shifts the antibody ensemble to promote hFcγRI binding. We further analyzed the cross-talk among subunits in the Aβ-solanezumab-hFcγRI complex. We found that Aβ binding and FcRs binding are highly correlated events. Not surprisingly, antigen binding signals are mainly transferred through the hinge region.^34^ These signals also propagate through the CL/CH1 domain as a bypass. These two pathways enhance the signaling from the antigen to FcR. We hypothesized that these allosteric events are entropy controlled. Antibody-antigen binding reduces the entropy of CDR loops. The entropy is transferred to the hinge region, leading to Fab re-orientation. Entropy may also be transferred to the Fc CH2 domains, leading to open CH2 domain conformations. CH2 transferred the entropy to the glycans, detached one heptasaccharide from the domain, facilitating hFcγRI recognition. Together, this work provides conceptual insight at the atomic level into the correlation of antibody-antigen recognition and effector function.

## Results

### Unbound antibody has highly dynamic conformational distribution; antigen binding shifts the population into two dominant clusters that facilitate FcR binding

**Figure 1.**
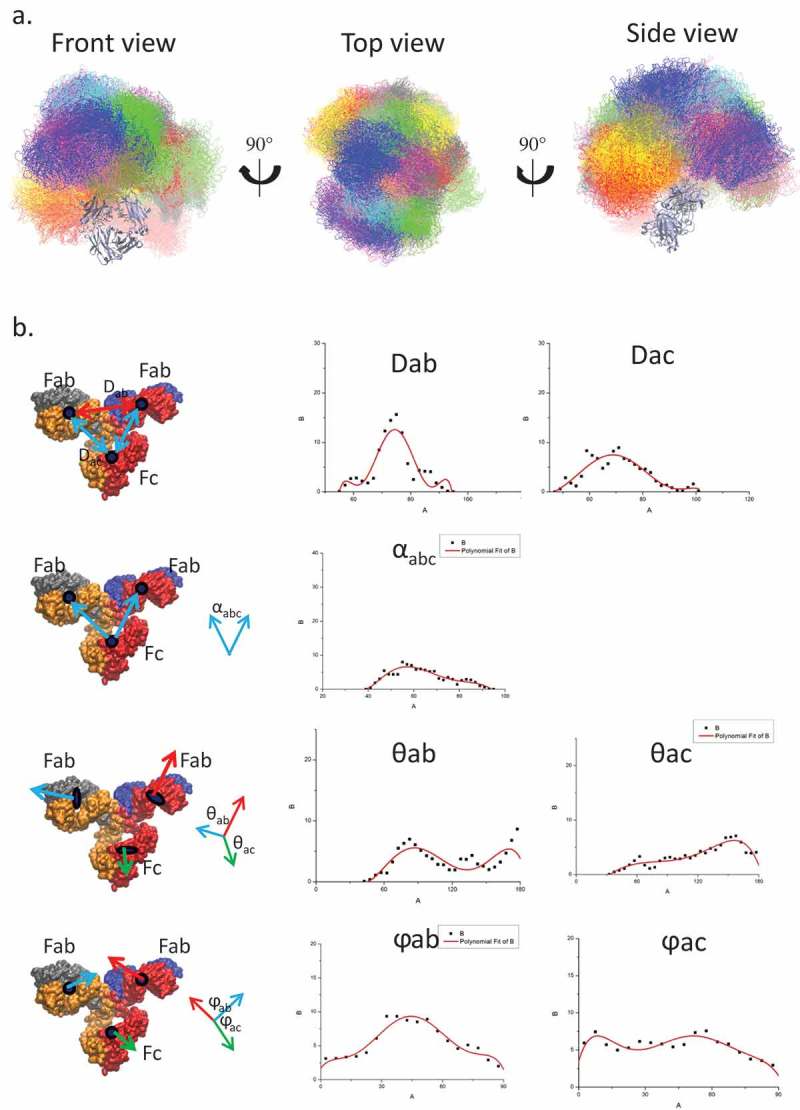
While the relative orientations of two Fabs and Fc domains cover a wide range of space, the distances between two Fabs have a Gaussian distribution narrower than that of Fab to Fc domain, suggesting that two Fabs may have correlated motions. a. Conformational ensemble obtained from the simulation covers a wide range of continuous space. The conformers from 12 independent runs are superimposed on the Fc domain and labeled in different colors. b. The distribution of center of mass distance (D_ab_: distance between two Fabs; D_ac_: distance between Fab and Fc domain), center of mass angle (α_abc_), sub-domain angles (θ_ab_: angle between two Fabs; θ_ac_: angle between Fab and Fc domain), and sub-domain plane normal angles (φ_ab_ and φ_ac_) of the unbound antibody.

As a first step, we simulate the conformational distribution of a free antibody in solution. To enhance the sampling, we performed 12 independent MD simulations of an unbound antibody with 12 different initial conformations (Figure 1a), including experimental structures of human IgG1 (1HZH), and murine IgG1 (1IGY). The results showed that the sampled conformations reach a wide range of space with reasonable overlap among the 12 MD simulations. This indicates that our simulations sampled an ensemble capable of adequate evaluation of the antibody space. We measured the domain center of mass (COM) distances, angles, and dihedrals and compared the distribution with the electron tomography (ET) data.^35^ A total of 160,000 structures were evaluated, and the distribution showed profiles similar to ET (Figure 1b). This suggested that our simulation ensemble represents the essence of the conformational distribution of the unbound antibody. Whereas the domain angles/dihedrals are widely distributed, the COM distance, especially the distance between two Fabs (D_ab_) concentrated at between 65 to 85 Å.

Even though our simulations and experimental ET provided similar profiles of the domain conformational distributions, the simulations attain higher resolution. For example, ET showed almost overlapping COM distributions for the distance between two Fabs (D_ab_) and between Fabs and Fc domain (D_ac_), whereas the simulation indicated that D_ab_ has a narrower distribution than D_ac_, suggesting that two Fabs do not move independently. The averaged contact area between the two Fabs (1118.1 ± 317.6 Å^2^) is larger than the contact area between Fab and Fc (848.1 ± 319.7Å2). Therefore, while the antibody is highly flexible and the three subunits (two Fabs and Fc) form/break contacts dynamically, the domains’ movements are not random.

**Figure 2.**
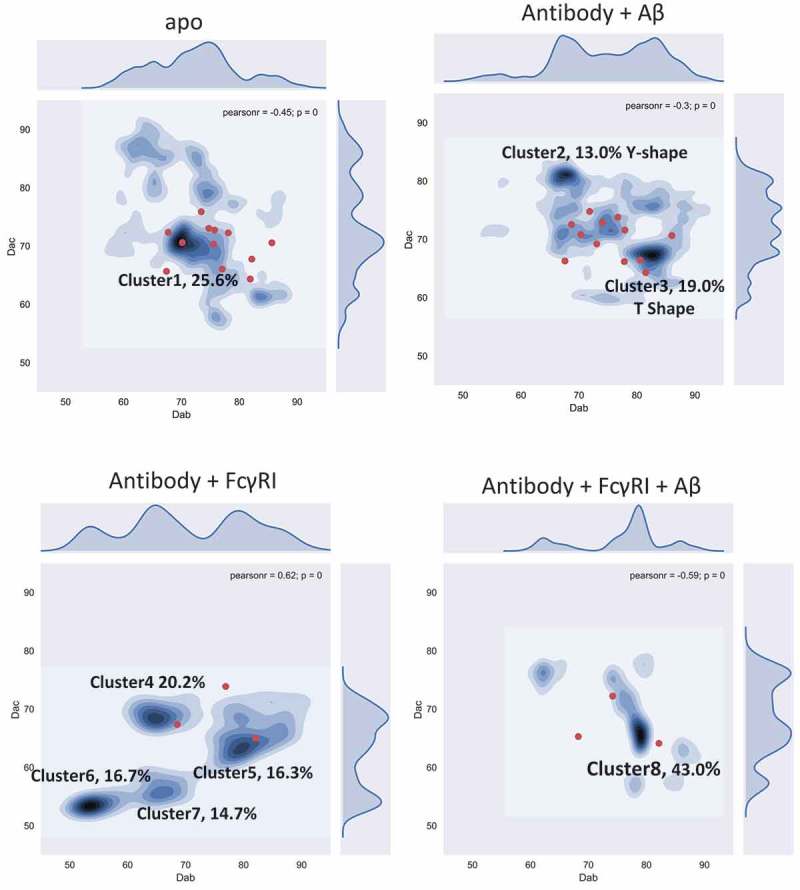
Conformational population redistribution of the antibody upon Aβ and hFcγRI binding indicates that antigen binding results in a uniform distribution of the population of the antigen•antibody•Fc-Receptor complex. The population is represented by the distribution of the center of mass distance between Fabs (D_ab_) and Fab and Fc (D_ac_) in the four complexes. The D_ab_/D_ac_ value of the initial conformation is represented in red dot. In cluster2, the Fabs-Fc distance is larger than Fab-Fab (Y shape), while in cluster3, the Fab-Fc distance is smaller than Fab-Fab (T shape).

Figure 2 shows the two-dimensional density distributions of the conformations obtained from MD simulations of the four systems. For the free antibody, the highest density (over 25% of the population) is around equal distance among Fabs and Fc domain (D_ac_ = D_ab_ = 70 Å), which could be the reason why it is hard to distinguish between D_ac_ and D_ab_ experimentally. However, when Fab is loaded with antigen, the conformational distribution changes dramatically. The original (D_ac_ = D_ab_ = 70 Å) cluster becomes less populated, while two major clusters centered around (D_ac_ = 65 Å; D_ab_ = 80 Å) and (D_ac_ = 80 Å; D_ab_ = 65 Å) appear. These two clusters, cluster2 and cluster3, contain ~ 13% and ~ 19% of the population. Their potential energies are very similar, although cluster2 is ~ 50 kcal/mol lower than cluster3. In cluster2, the Fabs-Fc distance is larger than Fab-Fab (Y shape), while in cluster3, the Fab-Fc distance is smaller than Fab-Fab (T shape).

We also explored whether the antibody can bind the Fc receptor without binding antigen. We simulated the antibody/hFcγRI complex (Figure 2). There are four major clusters and the populations become more separated. The four clusters have very similar potential energy: −24,862.4 ± 603.0 (cluster4), −24,827.9 ± 621.8 (cluster5), −24,884.3 ± 614.2 (cluster6), and −24,899.6 ± 657.4 (cluster7) kcal/mol. Two clusters, cluster6 and cluster7, do not appear in the unbound antibody distribution. Cluster5 has a similar profile compared to cluster3 in the antibody-antigen complex. Overall, the equal distributions of the four clusters suggests that the functional consequences of the antibody/hFcγRI complex without the antigen are not well defined.

For the antibody-FcR complex after antigen loading (antibody/antigen/hFcγRI), cluster8 dominates, with over 43% of the conformation concentrated around D_ac_ = 65 Å; D_ab_ = 80 Å). Cluster8 overlaps cluster3 in the antibody-Aβ system, close to cluster5 in the antibody/hFcγRI complex. Cluster8 could correspond to the antibody functional activation. Overall, the population shifts reveal that allosteric signaling can be transmitted through antibody conformational dynamics.

### Fc domain responds to antigen binding by increasing the population with open CH2

**Figure 3.**
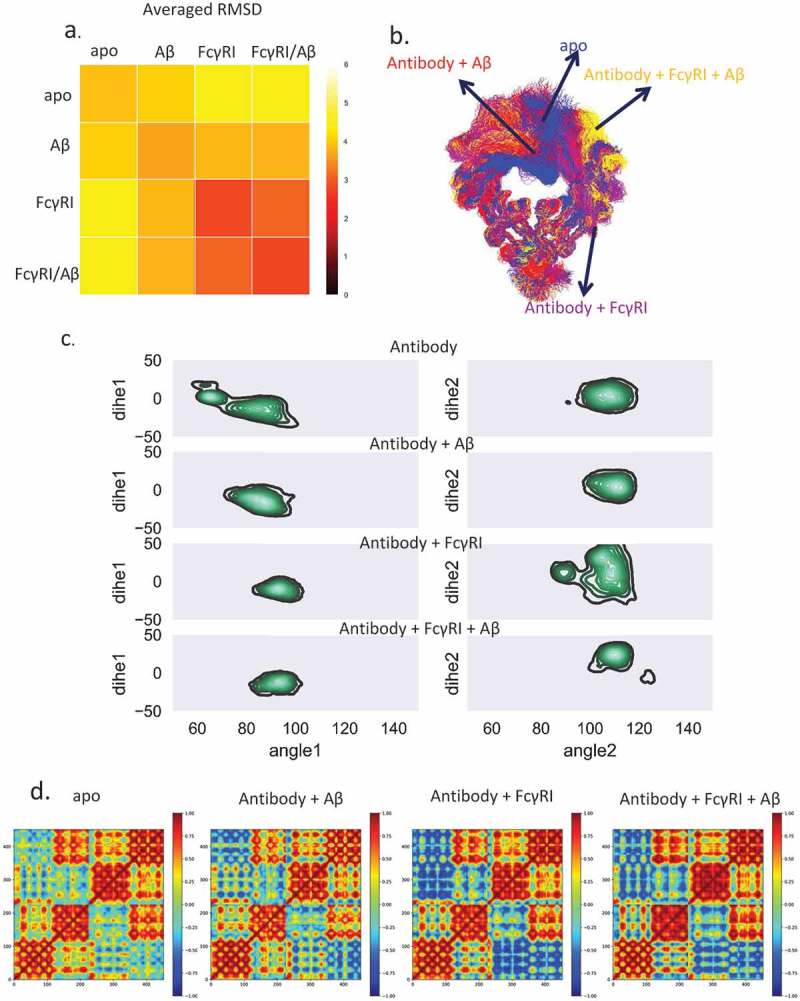
Dynamic motions in Fabs and Fc domains are correlated, and Aβ binding shifts Fc to open conformations to facilitate hFcγRI binding. a. The averaged RMSD among the four complexes indicates that the antigen•antibody•Fc-Receptor complex has a more uniformed conformational distribution. Each structure from each complex was compared to all other structures and averaged by root mean square deviations (Å). b. The most populated clusters from four complexes: unbound antibody (blue), antibody-Aβ (red), antibody-FcγRI (purple), and antibody-FcγRI -Aβ (yellow). c. Two-dimensional histograms show the distributions of the Fc CH2/CH3 relativeangle and dihedral angle. The population distribution from all available MD simulations are shown as contours. Three point angles were defined from the C atoms of residues Y514(1175), M642(1303), and Q576(1294) for the CH2/CH3 angle and four point dihedral angles were defined from the C atoms of residues Y514(1175), Y533(1194), M642(1303), and Q576(1294) for CH2/CH3 dihedral angles. Residue numbers in the brackets are the corresponding residues of the antibody heavy chain. d. Motion correlation among the residues of the Fc region of the four complexes indicated that dynamic motions in Fabs and Fc domains are correlated. Residues with highly (anti)correlated motion are red (blue).

We superimposed the CH3 domain and calculated the root mean square deviation (RMSDs) of the Fc region from the conformers obtained in the simulations of the four complexes. Each of the 48,000 structures was compared with all others. We averaged the RMSDs from each of the four complexes and averaged the RMSDs from two different complexes (Figure 3a). The 2D RMSD plot suggested that the Fc structures from the unbound antibody are very different from the hFcγRI-bound antibody, while Fc structures from antibody bound only to an Aβ antigen were similar to both unbound and hFcγRI-bound antibodies. This suggested that Aβ antigen binding induces Fc conformational changes toward conformations facilitating hFcγRI binding.

We clustered the Fc region using RSMD of 4 Å (Figure 3b). For the unbound antibody, 57.0% of the total 160,000 structures are in cluster1, while 18.1% are in cluster2. In cluster1, one subdomain of CH2 blocked the FcR binding sites, while in cluster2, this sub-domain adopted an open conformation. For the Aβ antigen-loaded antibody, over 73% of the population falls into one cluster. This suggested that antigen binding shifts the antibody conformations from cluster1 to cluster2. This cluster resembles cluster2 in the unbound antibody, with open conformation ready for Fc receptor binding. When hFcγRI bound to the antibody, whether Aβ-bound or unbound, there is only one dominant (both 87.8%) Fc region cluster. This suggested that the Fc region of the Aβ-bound antibody formed an intermediate conformation between the unbound and FcR-bound antibody.

The open Fc conformation is accompanied by the change of twist between CH2/CH3 of the Fc domain. We calculated the relative angle and dihedral angle for CH2/CH3 of the left and right chain in the Fc domain (Figure 3c). In the apo form, the left chain showed wide-spread distribution of both angles for the CH2/CH3 of the left chain and narrow distribution of right chain. In the antigen-bound case, the angles’ distribution concentrated on (75 ~ 100, −40 ~ 10) of the left chain and (100 ~ 120, −15 ~ 20) of the right chain. The angles’ space further narrowed when both Aβ and hFcγRI bind. For hFcγRI-antibody without antigen, the distribution of the right chain is more spread out.

The increase in the open Fc conformations expanded the exposure of the Fc receptor-binding residues as well. We defined Fc interfacial residues with contact frequency larger than 20% as key binding residues in Fc receptor recognition. The accessible surface area (ASA) of the key binding residues in the unbound antibody is 2333.5 ± 195.3 Å2, whereas in the Aβ-bound antibody it is 2449.5 ± 188.4 Å2, an ~ 116 Å2 increase upon Aβ binding. This suggests that Aβ binding increases the accessible area of Fc, which facilitates hFcγRI binding. The dynamic cross-correlation matrix (DCCM) of the Fc region of the four complexes (Figure 3d) indicated that in the unbound antibody, there is a weak correlation with the other CH2 sub-domain or CH3 sub-domain. In the hFcγRI-bound antibody, the correlation becomes negative, indicating the two CH2 sub-domains are apart following FcR binding. In the Aβ-bound antibody, although there is no hFcγRI binding, the CH2 sub-domains are also negatively correlated. Together, the results suggest that Aβ binding may induce structural and dynamic changes in Fc region that facilitate the hFcγRI binding.

**Figure 4.**
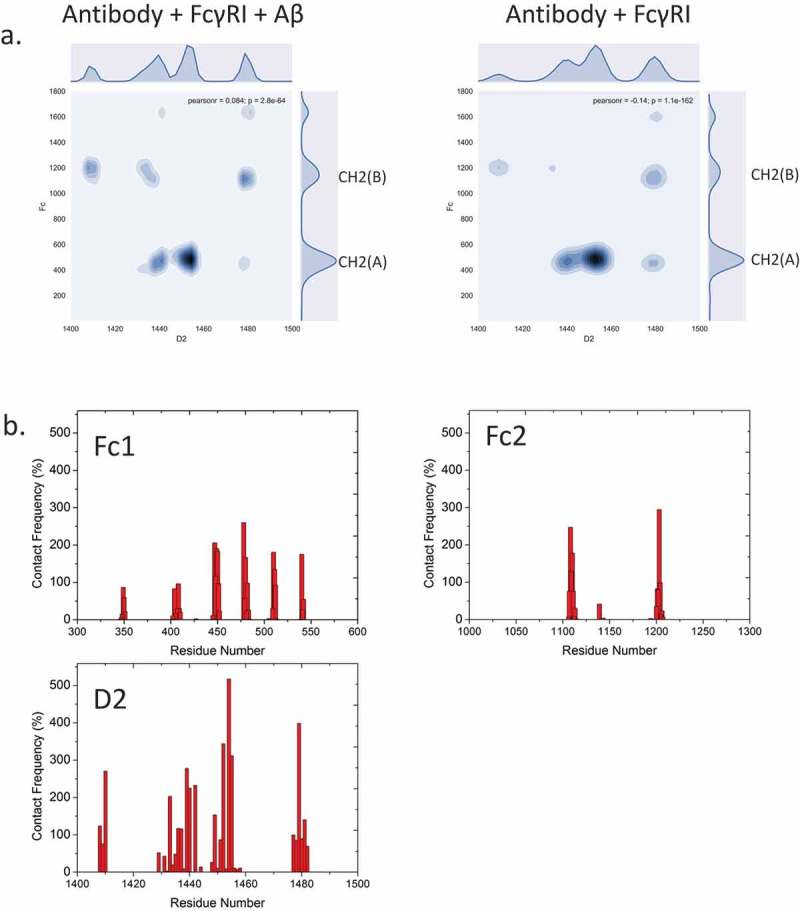
Fc-hFcγRI interactions and interfacial residues present asymmetric distributions of intermolecular contacts. a. 2D contact frequency map between antibody and Fc receptor D2 domain. b. contact frequency of residues from Fc1, Fc2, and hFcγRI D2 domain, respectively.

To verify that antigen binding changes the antibody conformation and dynamics, rather than direct interactions between Fc domain and receptor, we examine the electrostatic and hydrophobic interactions and the hydrogen bonds that are crucial for Fc/FcR recognition. We firstly evaluated the interfacial residues/glycans within 3Å between hFcγRI and antibody. We found that the patterns are similar with/without Aβ binding (Figure 4a). This suggested that direct hFcγRI and Fc recognition is independent from Aβ antigen-Fab recognition. The contact map showed asymmetric distribution between CH2(A) and CH2(B) sub-domains. Most interfacial residues formed both electrostatic and hydrophobic interactions (Table 1, Figure 4b). For example, the salt bridges of His1455-Asp479, Lys1452-Glu483, Lys1480-Glu1108 last 96.4%, 76.2%, and 75.5% of the total simulation time. Hydrophobic interactions of Trp1411-Pro1204, Met1478-Leu448, Tyr1440-Leu448 last 71.7%, 70.6%, and 68% of the total simulation time. Besides the Fc region, the Fab region, especially the CH1-1 loop, forms interactions with the hFcγRI D1 domain. For example, Ser350 forms hydrogen bonds withSer1345 and His1378 for 31.9% and 25.4% of the total simulation time. Gly351 and Gly352 form hydrogen bonds with Gln1334 for 22% and 20.6% of the total simulation time. In addition to the interactions already described, the glycan-residue interactions also play an important role. For example, β-N-acetylglucosamine (BGLCNA1631) form hydrophobic/aromatic interaction with Leu1443(75.4%) and Phe1453(72.6%). BGLCNA1631 also form hydrogen bonds with Arg1482 (61.2%) and Asn1441(49%).

**Table 1.** Interfacial residue pairs with > 20% intermolecular contact frequency between Fc domain and hFcγRI.

1RESID #1	RESNAME	RESID#2	RESNAME	(%)
1455	HIS	479	ASP	96.4
1452	LYS	483	GLU	76.2
1480	LYS	1108	GLU	75.5
1443	LEU	1631	D-glucose	75.4
1453	PHE	1631	D-glucose	72.6
1411	TRP	1204	PRO	71.7
1478	MET	448	LEU	70.6
1434	TRP	1204	PRO	69.6
1455	HIS	481	SER	69.2
1440	TYR	448	LEU	67
1482	ARG	1631	D-glucose	61.2
1440	TYR	449	LEU	56
1456	TRP	543	PRO	54.9
1440	TYR	1109	LEU	53.7
1411	TRP	1203	LEU	52.7
1456	TRP	449	LEU	50
1438	LEU	1109	LEU	49.4
1441	ASN	479	ASP	49.1
1441	ASN	1631	D-glucose	49
1480	LYS	1140	ASP	40.3
1482	ARG	1632	D-glucose	35.4
1345	SER	350	SER	31.9
1456	TRP	542	LEU	27.8
1434	TRP	1203	LEU	25.6
1378	HIS	350	SER	25.4
1455	HIS	484	ASP	25
1334	GLN	351	GLY	22
1482	ARG	479	ASP	21.9
1432	HIS	409	THR	21.7
1481	HIS	1646	α-D-mannose	21.3
1334	GLN	352	GLY	20.6

1. The RESID is the residue numbering in the simulation system. Please see supplementary in detail about numbering system.

### N-glycan and Fc conformational changes are synchronized following antigen binding

**Figure 5.**
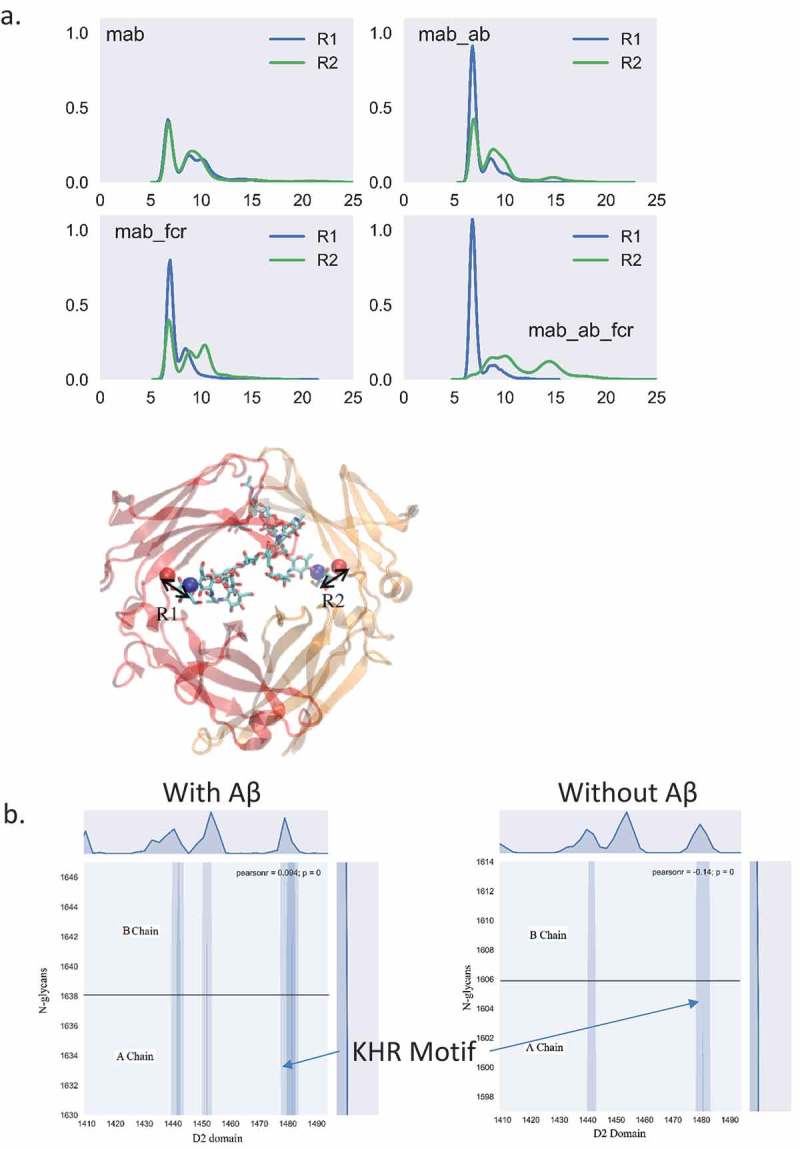
The N-glycan from both chains exhibit different dynamic behavior upon Aβ or FcγRI binding, indicating the importance of the N-glycan in allosteric signal transduction. (a) The distance between the C1 atom of (α1-6Man-linked) Gal and the Cα atom of proline 458 (left) and proline 1119 (right) are colored by blue and green, respectively. (b) Contact map between N-glycans and the D2 domain of FcγRI in the complex with and without Aβ binding.

Figure 5a shows the distance between the glycan tip and the nearby amino acid from the CH2 domain. In the apo form of the antibody, the distance showed similar distribution, including two major peaks at ~ 6 Å, and ~ 8 Å, corresponding to the bound and free states, respectively. In the apo antibody, the density distribution of the two N-glycan arms shows similar distributions, with the free state peak being slightly higher than the bound state peak. This result agreed with the work of Frank et al,^25^ in which only the Fc domain of the antibody was studied. However, when Aβ binds to the antibody, the distance distribution differs for the two sides of the antibody. The 6 Å peak of the left chain was enhanced while the distribution of the right chain remained similar to the apo form. This suggested that when Aβ binds to the antibody, one N-glycan arm showed dominant bound state conformation while the other arm showed both free and bound states. When hFcγRI binds to the antibody without antigen, the distance distribution showed a similar pattern to the antibody-Aβ complex. When both Aβ antigen and hFcγRI bind to the antibody, this effect is clearer. The 6 Å peak dominates the left chain, but there are two major peaks at ~ 10 Å and ~ 14 Å for the right chain, suggesting that the left glycan binds tightly to the Fc domain and the right glycan disassociates. Thus, when both Aβ and hFcγRI bind to the antibody, one N-glycan arm showed dominant bound state conformation while the other arm showed dominant free states.

In the complex with Aβ binding, the KHR motif (residue number Lys1480, His1481, and Arg1482) showed the largest contact area with one arm of the N-glycans with numerous hydrogen bonds (Figure 5b). Asn1441, Leu1443, Tyr 1445 and Phe1452 also contact the other arm of the N-glycans, but the intensity is lower. Without Aβ, these hFcγRI-N-glycan contacts decreased, altogether suggesting that Aβ binding induced glycan conformational changes that facilitate the hFcγRI binding.

### Limited signaling through the residue contact network from FaB to the Fc receptor

The structural changes of the paratope and variable domain were analyzed (Fig. S5). The VH/VL orientation fluctuation decreased after Aβ binding (Fig. S5a), with the RMSDs of the paratope (Fig. S5b), i.e., each individual CDR loop, showing lower structural flexibility. The root mean square fluctuations (RMSFs) of individual residues in the variable domain also suggested that the CDR loops diminished their flexibility. We also found that the RMSFs of non-CDR loops also decreased upon antigen binding. This suggests that the non-CDR loops of the variable domain, which is not directly in contact with the antigen, respond to the antigen binding.

**Figure 6.**
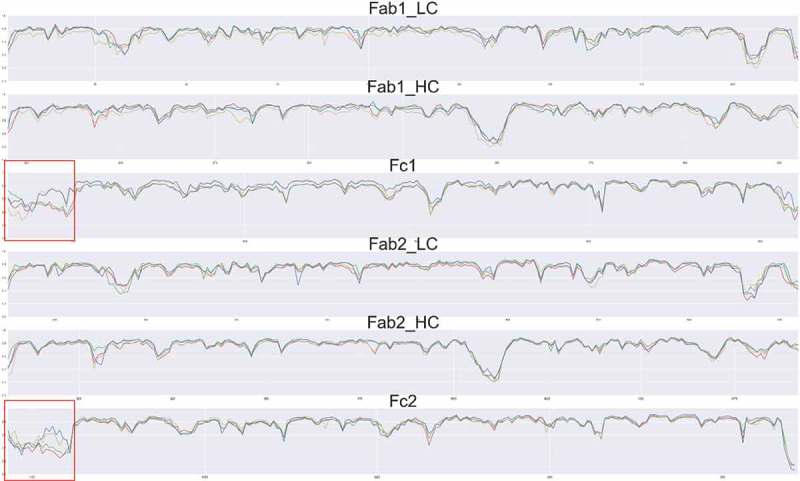
Order parameters S^2^ of the antibody in the four complexes indicate change in residue flexibility of the antibody with and without antigen binding. The values for the unbound antibody, antibody-Aβ, antibody-FcγRI, and antibody-FcγRI-Aβ are colored red, green, yellow and blue, respectively. The hinge region is highlighted by a red square.

Sharp et al. found that protein backbone entropy and order parameters obtained from MD simulations are correlated.^36^ To evaluate all residues, including proline, we calculated the generalized order parameter S^2^ of the C = O bond of each individual residue of the antibodies (Figure 6). The averaged S^2^ of the C = O bond of the CDR region for the Aβ-bound antibody has higher order parameter than without Aβ. For the hFcγRI-bound antibody, the CH2 region showed higher order parameter than without hFcγRI. Thus, Aβ and hFcγRI binding reduce the local entropy.

The hinge region, which connects the Fabs and the Fc region, showed the largest S^2^ change among the four complexes. When hFcγRI binds to Fc, the order parameter increased ~ 50% compared with the unbound antibody, mostly due to contact between hinge residues and the Fc receptor. When Aβ binds to the CDR region, although the CDRs are distant from the hinge, the order parameter in the hinge region increases by ~ 20% increase. As the hinge region rigidifies (high order parameter), allosteric signaling from Aβ to hFcγRI directly through amino acid residue contact network could be more efficient.

**Figure 7.**
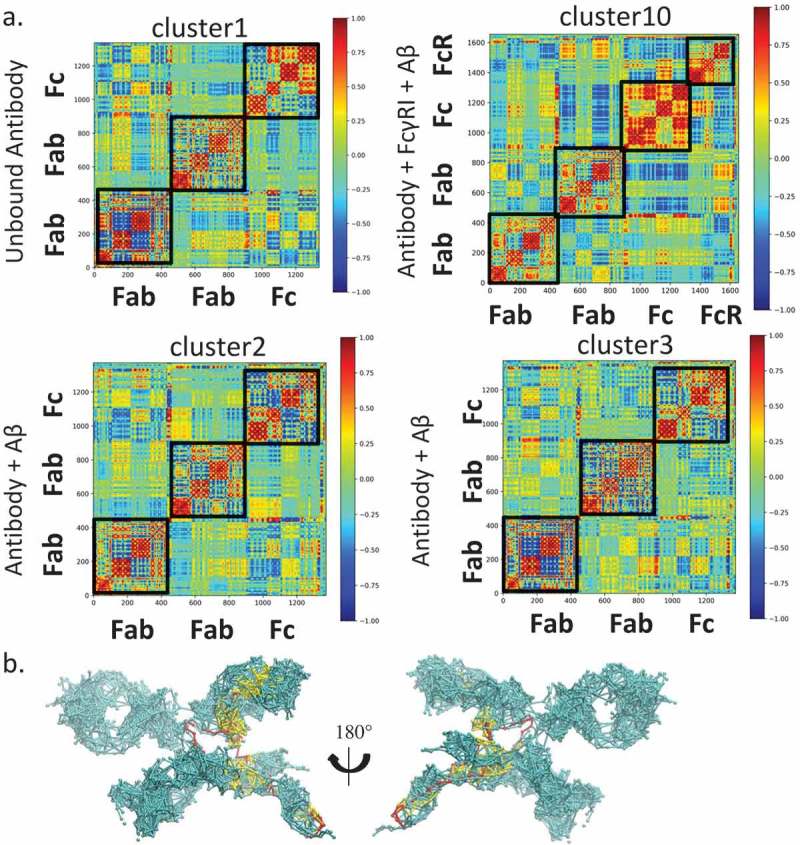
Allosteric Fab-FcγRI communication is via limited residue contact pathways, highlighting the importance of conformational population shift in allosteric signal transduction. a. Motion correlation among residues of different clusters. Residues with highly (anti)correlated motion are red (blue). The cluster numbers correspond to the clusters in Figure 2. b. Optimal and suboptimal paths connecting plausible allosteric sites from Aβ to FcγRI D3 domain in the Fab-FcγRI- Aβ complex (cluster10). Optimal and suboptimal paths are colored by red and yellow, respectively.

We examined if, upon Aβ binding, the Fab can directly transfer the signals to hFcγRI through the residue contact network. In the antibody- hFcγRI-Aβ complex, we found that the population converged to a dominant cluster (cluster10) with ~ 43.0% of the total population. We obtained all the structures in this cluster and analyzed the subdomain communication and signaling from Aβ to hFcγRI. Evaluation of the DCCM of the whole complex indicates that there is positive correlation between the Fab or Fc region or hFcγRI (Figure 7a). In contrast to the antibody-Aβ-hFcγRI complex, the unbound antibody showed low motion correlation among Fabs and Fc. This suggested that once bound with hFcγRI, the antibody motion become synergistic. In cluster2 of the antibody-Aβ complex, Fabs and Fc showed similar low motion correlation like the unbound antibody, while in cluster3 of the antibody-Aβ complex, Fabs and Fc showed stronger motion correlation compared with cluster3 and cluster1 in the unbound antibody.

We also used the community network analysis, and considered the antibody as a network to evaluate the communication among the domains (Figure 7). Nodes within the community communicate more frequently than nodes outside the community. In the unbound antibody, there are 12 communities in Fabs and Fc, corresponding to the subdomains (Fig. S4). The two hinge chains form two independent communities. In the antibody-Aβ complex, either in cluster 2 or cluster3, the CH1 and CL merged into a single community, suggesting that Aβ binding reorganized the community. In the antibody-Aβ-hFcγRI complex, the two CH3 domains merged into one community while one hinge loop and CH1 of hFcγRI formed another. Thus, binding of either Aβ or hFcγRI enhance the communication between subdomains. Analysis of pathways from Aβ to hFcγRI through the antibody (Figure 7b) indicates that the shortest pathway bypasses the hinge region, while many suboptimal pathways go through the Fab constant domain, especially from the CH1-1 loop, directly to hFcγRI. The suboptimal paths are 4 ~ 5 longer than the optimal path, providing alternative pathways from Aβ to hFcγRI. When only hFcγRI binds, we identified four clusters. In these clusters, there are almost no contacts between CH1-1 loop and hFcγRI, and thus no pathways through this region (data not shown).

**Figure 8.**
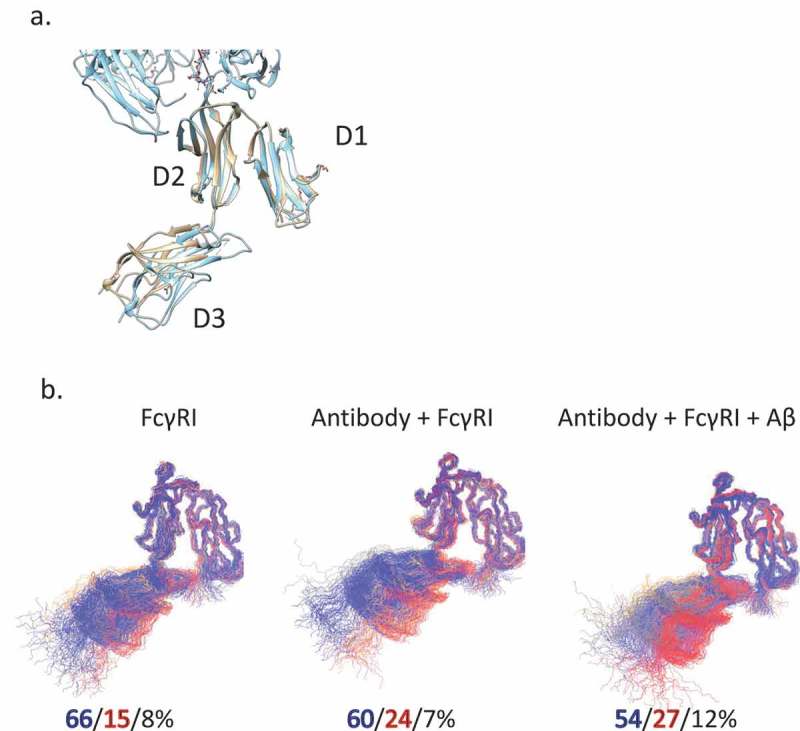
FcγRI conformational distributions may also reflect antigen binding. a. Superimposed crystal structures of FcγRI(pink) and Fc-FcγRI complex(cyan). b. The clustered structures of FcγRI in their apo form, bind to free and Aβ-bound Fc. The top three clusters are colored in blue, red, and gray, respectively.

The final question to be answered is if the Fc receptor conformational dynamics can show different signals from binding of antibody with and without antigen. To evaluate the motion and conformational change of hFcγRI, we superimposed the D1, D2 domain as they bind to the Fc domain. We measured the angles between neighboring domains (Figure 8a). In the crystal structures, the angle between D2 and D3 in Fc-hFcγRI is ~ 160°. We found that the angle between D1 and D2 is homogenous around 36°, but the angle between D2 and D3 fluctuates substantially. In bulk solution, the D3 domain is dynamic and there are two distinct clusters: the major cluster A (66%) with the D2-D3 angle of 140° and minor cluster B (15%) with an angle of 160°. When hFcγRI-bound, the population shifts slightly to cluster B (24%); when Aβ-bound, cluster B is at 27%.

## Discussion

Allostery is an intrinsic protein property,^37^ and allosteric signaling can be transferred through protein conformational dynamics and population shift.^37,38^ Conformational dynamics permits both promiscuity and specificity.^34,39–44^ Protein complex formation redistributes the dynamics,^45,46^ allowing allosteric signaling through protein domains. Antibody-antigen recognition, which is associated with structural transitions through inherent conformational flexibility,^5–7^ involves conformational selection.^47^ To regulate the immune response, antibody-antigen interaction sends a signal for complement activation and Fc receptor binding. Signaling pathways depend on the sequences of the variable regions, through hydrogen bonding network, electrostatic interactions, and residue contacts. Through their changes, they result in population shift of dynamic conformations.^48^ However, intramolecular signaling is complex and exactly how it takes place in distinct structures and under certain conditions is still not entirely clear.^14,32,33^

The Fab variable domain recognizes the antigen, followed by effector activation by the Fc domain. Classically, these two processes were thought to be independent. This led to the associative model in which antigen (largely)-mediated crosslinking of Fab domains increases the proximity of the Fc domain, leading to higher avidity for FcγR and C1q.^49,50^ However, recent studies showed cross-talk between the variable and constant domains. For example, different IgG subclasses with identical V domains exhibit different target-binding affinities and specificities.^4,51,52^ Several studies have shown that modifications of the constant domain (e.g., disulfide bonds^52^) or altering the entire constant domain^15^ of a Fab can influence the antigen binding affinity. Antigen binding correlates with long range conformational change in the constant domain of Fabs, especially the CH1-1 loop.^17^ Our studies indicate that V domain recognition and C domain effector function are dependent on each other, which contradicts the classical “independent” theory.

How do these two processes take place synergistically? Molecular dynamics simulation and fluorescence anisotropy have shown that antibody molecules are highly flexible.^53^ Recent electron tomography of IgG1 antibody molecules showed similar flexibility, but the subdomains distance/angles were not evenly distributed.^35^ Coarse-grained modeling showed that the antigen binding process is highly related to the internal dynamics of the IgG.^54^ Our simulations generally agree with the Fab-Fab and Fab-Fc angles and distance^35,55^ of IgG1. Due to the different number of disulfide bonds and length of the hinge region, the distribution may vary in other IgG subtypes.^56^

We observed population changes with (sub)domain-(sub)domain distance when the antibody binds to antigen and/or hFcγRI. The entropy change from the antigen binding can be transferred to the hinge region, and then to the Fc CH2 domains, coupled with opening the Fc conformations to facilitate the Fc receptor binding. Conformational change of the Fc CH2 domains upon hFcγRI binding has been discovered by crystal structures.^57^ After hFcγRI binding, the distance between the two CH2 domains increased to 9.1 Å. Our simulations showed that in the dominant conformation of the unbound antibody, the Fc CH2 subdomains are close to each other. In the dominant conformation upon Aβ binding, the Fc CH2 subdomains are more open. The structures become similar upon Fc binding to hFcγRI. Thus, antigen binding changes the Fc domain to an intermediate conformation between the unbound and hFcγRI-bound states. It has been reported that the hinge region and the CH2-CH3 interface residues are important for CH2-CH2 motion and conformation,^25^ and that modification of the human IgG1 hinge region can modulate its effector functions.^58^ The order parameters of the antibody hinge region suggested that the flexibility is reduced upon antigen binding. The change in flexibility in the hinge region further influences the CH2-CH2 motion, suggesting that the hinge region served as a linker as well as an “entropy transport cable” while transferring the antigen binding signals.

**Figure 9.**
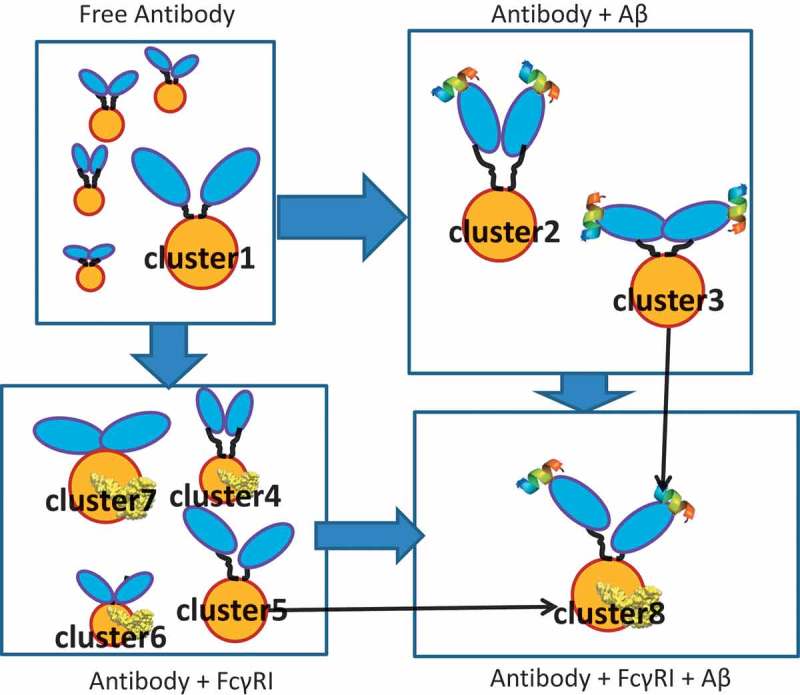
Antibody-antigen recognition mechanism. The two Fab domains are shown in blue, the Fc domain is shown in yellow, and the Aβ peptide is represented as helical. Cluster numbers correspond to Figure 2.

In addition to the CH2 conformational shift, we observed that antigen binding shifts the relative Fab-Fab and Fab-Fc orientation into two main clusters, one “Y”-shaped, the other “T”-shaped (Figure 9). In the available crystal structures of full-length antibodies, we observed both “T”-shape-like conformations, e.g., 1HZH, 1IGT and “Y”-shape-like conformations, e.g., 1IGY, 5DK3. Although these structures are from IgG1(1HZH, 1IGY), IgG2a(1IGT), and IgG4(4DK3), it seems that the two conformations are common among IgGs. The “T” shape conformation also exists in the antibody-Aβ-hFcγRI complex in our simulation, and likely represent the hFcγRI-bound structure. The “Y” shape conformation might surface upon binding to other partners, e.g., other Fc receptors and C1q. Some partners may prefer the “T” shape, while others the “Y” shape. For example, complement activity was augmented when the cognate antigens bind and a hexamer complex is formed. Transmission of allosteric signaling from the antigen-bound Fab to the Fc is essential for complement activation.^59^ We have proposed a general antibody-antigen recognition mechanism based on the population shifts, as illustrated in Figure 9.

The glycans at Asn-297 (N-glycan) help maintain the quaternary structure and Fc stability, ^60^ and thus Fc-FcR recognition.^61–64^ Deglycosylation of IgG1 resulted in a 40-fold loss in FcγRI binding.^61^ The noncovalent interactions of multiple Fc domain residues with the N-glycan are necessary for optimal recognition of FcγRI.^65^ Single amino acid mutations of these Fc residues affect glycan processing.^65–67^ In the apo form, terminal carbohydrate N-glycans residues are flexible: α1-3Man-linked branch is usually unconstrained, while the α1-6Man-linked branch has two states, free and bound to nearby Fc domain polypeptides.^68,69^ N-glycans dynamics are crucial in Fc-receptor interactions and enzymatic glycan remodeling.^68^ The composition of N-glycans can modulate the binding affinity of IgG1 Fc to FcγRs.^70–73^ X-ray crystallography and NMR data indicated that the two arms of N-glycan are either in the bound state (attached to the Fc)^74^ or in the free state (detached to the Fc).^68^ N-glycans may directly interact with the hFcγRI D2 domain (PDB ID: 4X4M),^61^ but do not show direct contact with hFcγRI in a high resolution structure (PDB ID: 4W4O).^57^ Based on our 6 independent MD simulations of hFcγRI-antibody complexes built on the high resolution structure (PDB ID: 4W4O),^57^ we confirmed that hFcγRI D2 domain, especially the KHR motif formed extensive hydrogen bonds with the N-glycans. Lee et al reported that the C’E loop and the CH2-CH3 orientation are dynamic, and changes in N-glycan composition optimize the interface with the Fc receptor. ^75^ In this study, we showed that the N-glycans also respond to antigen binding and shift their conformations and the CH2 domain ensembles. Antigen binding shifted one N-glycan arm to the bound state and the other to the free state. The asymmetric distribution of the states is similar to the distribution when hFcγRI binds. Thus, binding shifts the N-glycans to an asymmetric ensemble, which is required for hFcγRI binding.

The population shift mechanism described here suggests two-way communication between the Fc and Fv domains, i.e., modifications of Fc can influence the Fv antigen recognition. In line with this, IgA Fc mutations have been reported to reduce binding to human epidermal growth factor receptor 2 (HER2).^76^ Structural (circular dichroism,^77^ NMR,^78^ and crystallography^79^) data has shown that, upon antigen binding, the C domains can affect the V region paratope conformation. Simulations and experiments showed that modification of the constant domain influences binding affinity^80-82^ and specificity^83,84^ of the antibody-antigen interaction. This may have important implications in antibody engineering and isotype choice. Lua et al. used antibody isotype swapping by grafting the VL and VH of trastuzumab and pertuzumab onto human CHs and CLs to minimize side effects.^16^ They showed that the LC constant region changes have no major effects on HER2 binding, while some IgM and IgD heavy chain isotypes can modulate it. Xia et al. showed that the constant region plays an important role in the nephritogenicity of anti-DNA antibodies by affecting immunoglobulin affinity and specificity,^85^ with the order of IgG3> IgG2a> IgG1> IgG2b> IgM. Not all C domain (isotype) changes cause V region changes,^86^ suggesting a possible dependence on antigen type. Engineering is often done to reduce antibody size; however, with reduced size, entropy dissipation may be limited. In the case of the unstable scFv, only the V portion exists with the VH and VL domains connected by a linker; thus, stabilizing any CDR loop in the VH domain triggers a destabilizing response in all CDR loops in the VL domain and vice versa.^87^ The entropy upon antigen binding cannot be released to the C portion and might result in instability, as observed for the gammabody heavy chain variable domain, ^88^ with a long grafted CDR3 loop stabilized upon antigen binding, but limited ability to dissipate entropy.^89^ Aβ peptide rigidifies the solanezumab Fab domain,^15^ implying a high entropy penalty. However, within the full antibody framework studied here, we do not see a similar Fab domain rigidification. Therefore, even though the lack of C domain might not hinder affinity/specificity directly, it may limit it.

In conclusion, antigen recognition and FcR binding result in conformational change and subdomain cross-talk. The apo antibody is highly flexible, and its motion is not random.^34^ When bound to antigen, the relative Fab-Fab and Fab-Fc orientation shifts to dominant conformational clusters that may facilitate the FcR or C1q recognition, with the Fc CH2 domain becoming more open. We propose that population shift and the associated entropy redistribution is the major allosteric mechanism in antibody activation.

## Methods

### Molecular modeling and simulations

#### Systems construction

The sequences of solanezumab, hFcγRI, and Aβ are listed in Table S1. As the non-sequential Kabat numbering scheme is used in the crystal structures, we renumber the residues for convenience (see Supplementary file). The structures of the Fab/peptide complex were built based on the crystal structure Protein Data Bank ID 4XXD.^90^ To construct the Fc region, we performed sequence alignment of CH2/CH3 domains between solanezumab and 4W4O. There is only one residue difference (Fig. S1). Thus, the Fc region was directly built from the structure of 4W4O with mutation from alanine to serine. The N-glycans of solanezumab Fc and glycans of FcγRI were modeled directly from the corresponding templates (Fig. S2). Missing residues were modeled by template-based homology modeling using the SWISS-MODEL Server.^91^ To determine the relative positions of Fab and Fc within the full antibody, we used 1IGT as the template in which the distances between Fc COM and either Fab COM are roughly similar to avoid bias in initial configuration. The rebuilt systems (Fig. S3) were submitted to CHARMM-GUI glycan reader for the input for the MD simulation. The antibody-Aβ, antibody-FcγRI complex, and antibody was generated by removing FcγRI, Aβ, and FcγRI/Aβ, respectively.

#### Initial conformation generation and selection

Initial antibody random conformations were generated by adjusting three sets of torsion angles. 231C-232N-232CA-232C, 232N-232CA-232C-233N, and 232CA-232C-233N-233CA (numbering in 1IGT), each step with 60° rotation. During the conformation randomization, the Fc domain was fixed and the Fab domains move freely, leading to 216 conformations. Excluding conformations with closed Fab domain or with Fc domain clashes, 12 conformations were selected as the starting points for the simulations. In the complexes between hFcγRI and antibody, 4 of 12 representative conformations are selected to avoid clashes between Fabs and hFcγRI.

#### MD simulation protocols

Conserved disulfide bonds were constructed according to the specific IgG subtypes. The N-termini and C-termini were charged, NH_3_^+^ and COO^−^, respectively. The systems were solvated by TIP3P water molecules, and sodium and chlorides were added to neutralize the system and to achieve a total concentration of ~ 150 mM. The systems were energy minimized for 5000 conjugate gradient steps, where the protein was fixed and water molecules and counterions could move, followed by additional 5000 conjugate gradient steps, where all atoms move. In the equilibration stage, each system was gradually relaxed by a series of dynamic cycles, in which the harmonic restraints on proteins were gradually removed to optimize the protein-water interactions. In the production stage, all simulations were performed using the NPT ensemble at 310 K. All MD simulations were performed using the NAMD software^92^ with CHARMM36 force field.^93^ MD trajectories were saved by every 2 ps for analysis. A summary of all simulation systems is listed in Table S2.

#### Structural analysis

To calculate the VH/VL orientation, the two antibody structures, apo and Aβ-bound, were superimposed according to the variable domain of the H/L chains, and the RMSD is calculated. As the full-length antibody has two Fab domains, the VH/VL orientation was evaluated separately. The six CDRs were defined as described by Ofran et al.^94^ The RMSD was averaged over all pairs of either apo or Aβ-bound structures from all 12 MD simulations. The RMSFs were evaluated by the internal module of CHARMM.

#### Cluster analysis

To study the populations of the Fc domain, the trajectories of the four systems were aligned by the CH3 domain (residues 543 to 633 and 1207 to 1322), clustered by the Fc domain (443 to 633 and 1107 to 1322) using the clustering tool of VMD with cluster number of 5 and RMSD cutoff of 4 Å. To evaluate the conformational distribution of the full-length antibody, the trajectories of the four systems were aligned and the distance between the center of mass of two Fabs and between the center of mass of one Fab and Fc were measured and mapped onto a 2D plane.

#### Accumulated contact map

To identify the essential interactions between Fc and Fabs, all atoms within 3 Å between Fc and Fabs during the last 100 ns simulation were considered as input into PROTMAP2D,^95^ which can calculate the accumulated contact map by summing up all the frames during simulations.

#### Binding energy evaluation

To evaluate the total potential energy of the system, the trajectory for each system was extracted from the last 20 ns of explicit solvent MD without water molecules and ions. The solvation energies of all systems were calculated using the generalized Born method with molecular volume (GBMV)^96^ after 500 steps of energy minimization to relax the local geometries caused by the thermal fluctuations that occurred in the MD simulations. In the GBMV calculation, the dielectric constant of water is set to 80 and no distance cutoff is used.

#### Correlation analysis

Correlations between the residues in the different clusters from the four systems were analyzed using the normalized covariance to characterize the correlation in motion of protein residues,^97–100^ ranging from −1 to 1. If two residues move in the same (opposite) direction in most frames, the motion is considered as (anti-)correlated, and the correlation value is close to −1 or 1. If the correlation value between two residues is close to zero, they are uncorrelated. The correlation evaluation was performed using CARMA.^101^ The weighted network, optimal/sub-optimal paths in Fab/peptide systems is analyzed using NetworkView ^102^ module in VMD.

## Abbreviations

Aβ: Amyloid beta
ADCC: antibody-dependent cellular cytotoxicity
CDRs: complementarity-determining regions
COM: center of mass
ET: Electron Tomography
Fab: fragment antigen-binding
Fc: Fragment crystallizable region
FcR: Fc receptor
HC: heavy chains
hFcγRI: Fcγ receptors I
IgG: Immunoglobulin G
LC: light chains
RMSDs: root mean square deviations
RMSFs: root mean square fluctuations
TCR: T-cell receptor.
Fab: antigen-binding fragment
MD: molecular dynamics
V domain: antibody variable domain
C domain: antibody constant domain
VL: light chain variable domain
CL: light chain constant domain
VH: heavy chain variable domain
CH1: heavy chain constant domain-1
Sola: solanezumab
Cre: crenezumab
Fc receptors: FcRs

## Supplementary Material

Supplementary can be accessed here.
