# Supplementary material for: Antigen binding allosterically promotes Fc receptor recognition
Source: MAbs. 2018 Oct 5;11(1):58–74. doi: 10.1080/19420862.2018.1522178 (PMC6343797; doi:10.1080/19420862.2018.1522178)
Supplement: Supplemental Material [file kmab-11-01-1522178-s001.docx]

Antigen binding allosterically promotes Fc receptor recognition

**Jun Zhao^1^, Ruth Nussinov^2,3^, Buyong Ma^2^***

^1^Cancer and Inflammation Program, National Cancer Institute, Frederick, Maryland 21702

^2^Basic Science Program, Leidos Biomedical Research, Inc. Cancer and Inflammation Program, National Cancer Institute, Frederick, Maryland 21702

^3^Sackler Inst. of Molecular Medicine, Department of Human Genetics and Molecular Medicine, Sackler School of Medicine, Tel Aviv University, Tel Aviv 69978, Israel

* Corresponding author: E-mails: [mabuyong@mail.nih.gov](mailto:mabuyong@mail.nih.gov)

Running title: Allosteric signaling between antigen and Fc receptor


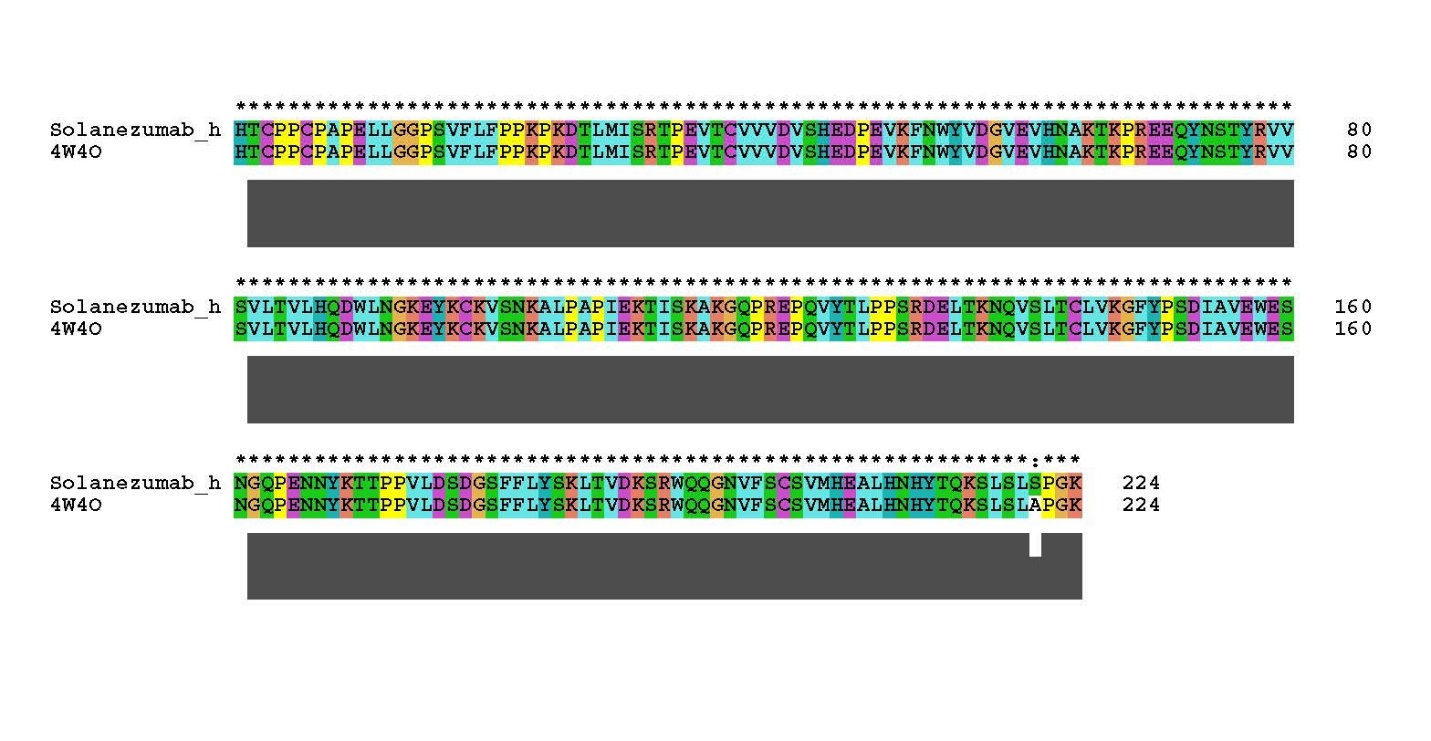
Supplementary figures and tables

Fig. S1 Sequence alignment of the CH2-CH3 part of solanezumab and 4W4O.


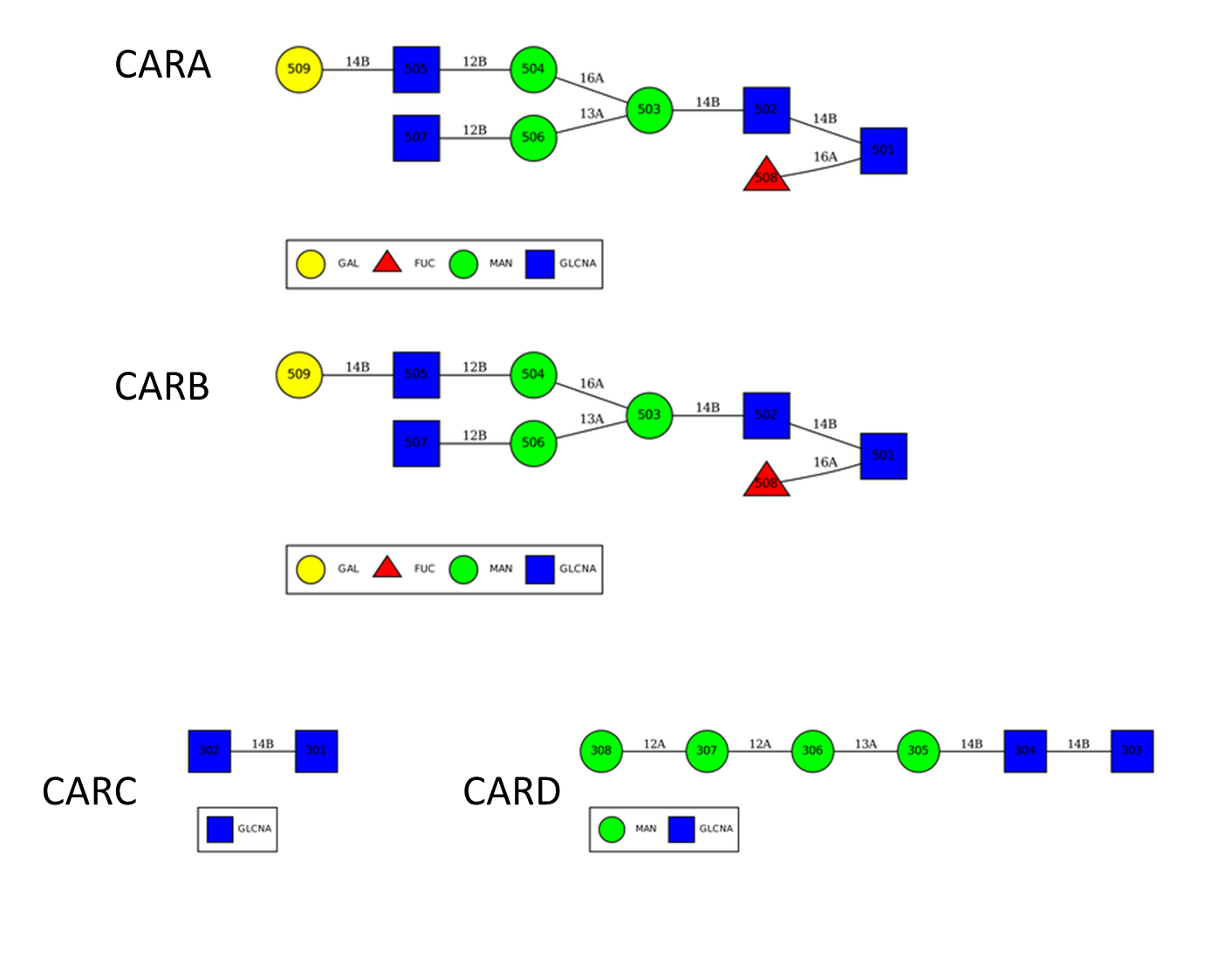
Fig. S2 Glycoforms of IgG1 Fc and hFcγRI used in this study. Both Fc polypeptides have identical glycans. CARA and CARB are N-glycans from the antibody while CARC and CARD are glycans from hFcγRI. Color code: blue square: N-acetylglucosamine, green circle: mannose, red tiangle: fucose, and yellow circle: galactose.


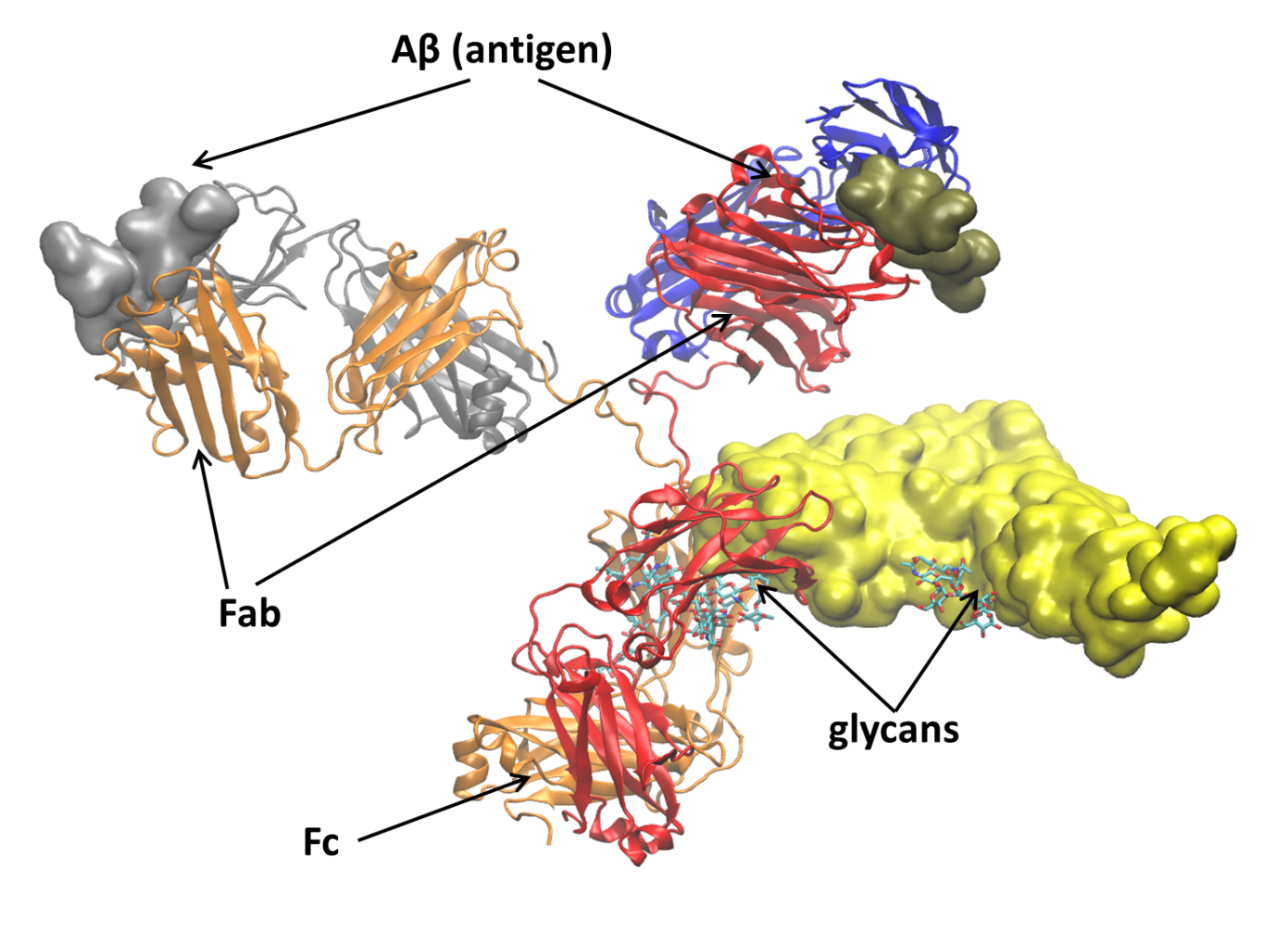
Fig. S3 3D structure of the Aβ-antibody-hFcγRI complex. The light chains of the antibody are colored by gray and blue, the heavy chains of the antibody are colored by orange and red, Aβ peptides are represented in VDW model colored by gray and tan, the hFcγRI is colored in yellow, and the glycans are represented in licorice.


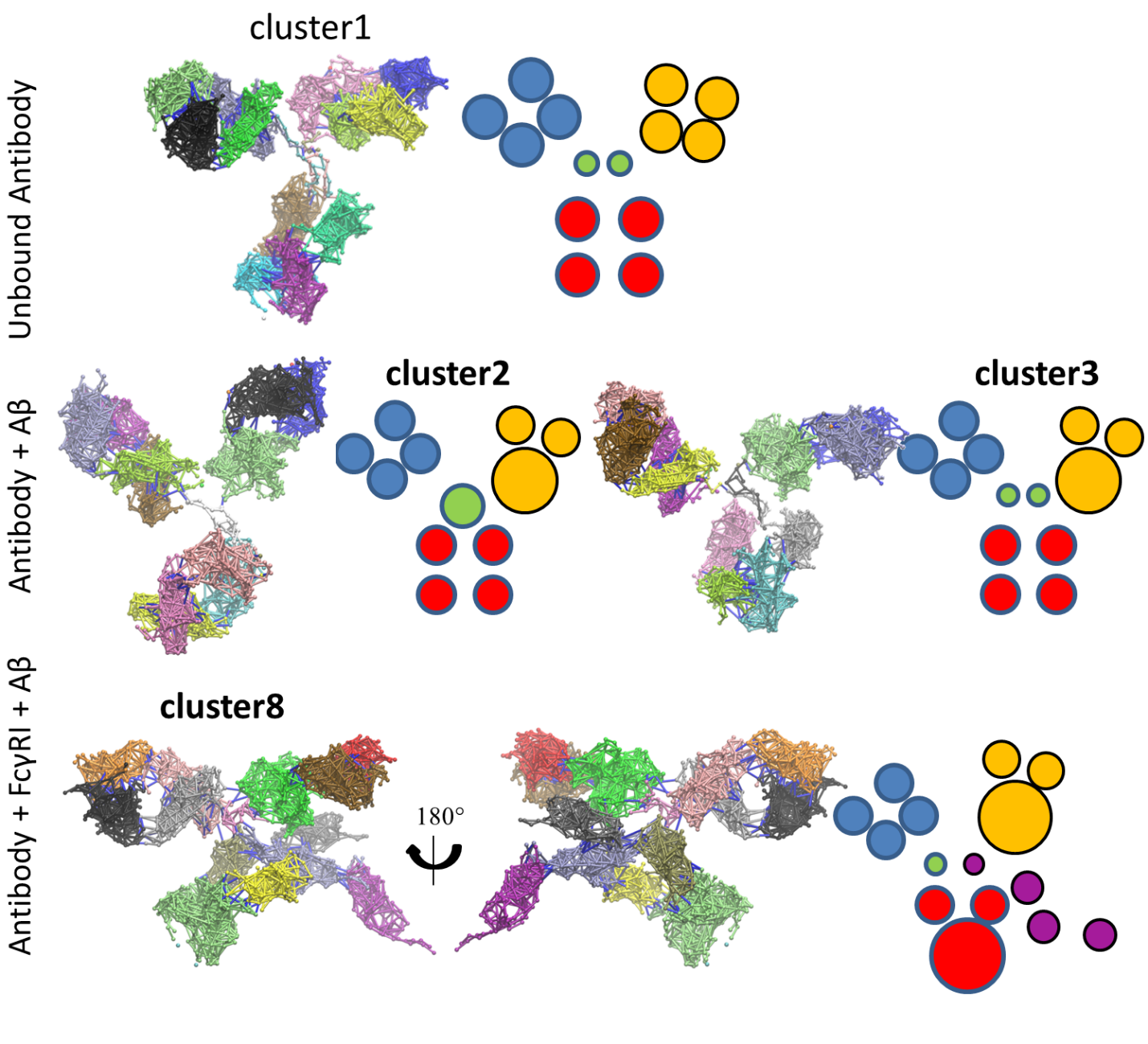
Fig. S4. Community analysis of the network constructed base on the motion correlation analysis of Fig. 6. Community distribution on the complex is showed on left panel and residues belong to the same community are in the same color. The corresponding cartoon is on the right panel. In the cartoon, one circle represents one community, and the communities from Fab1, Fab2, hinge region, Fc, and FcR are colored by blue, yellow, green, red, and purple respectively.


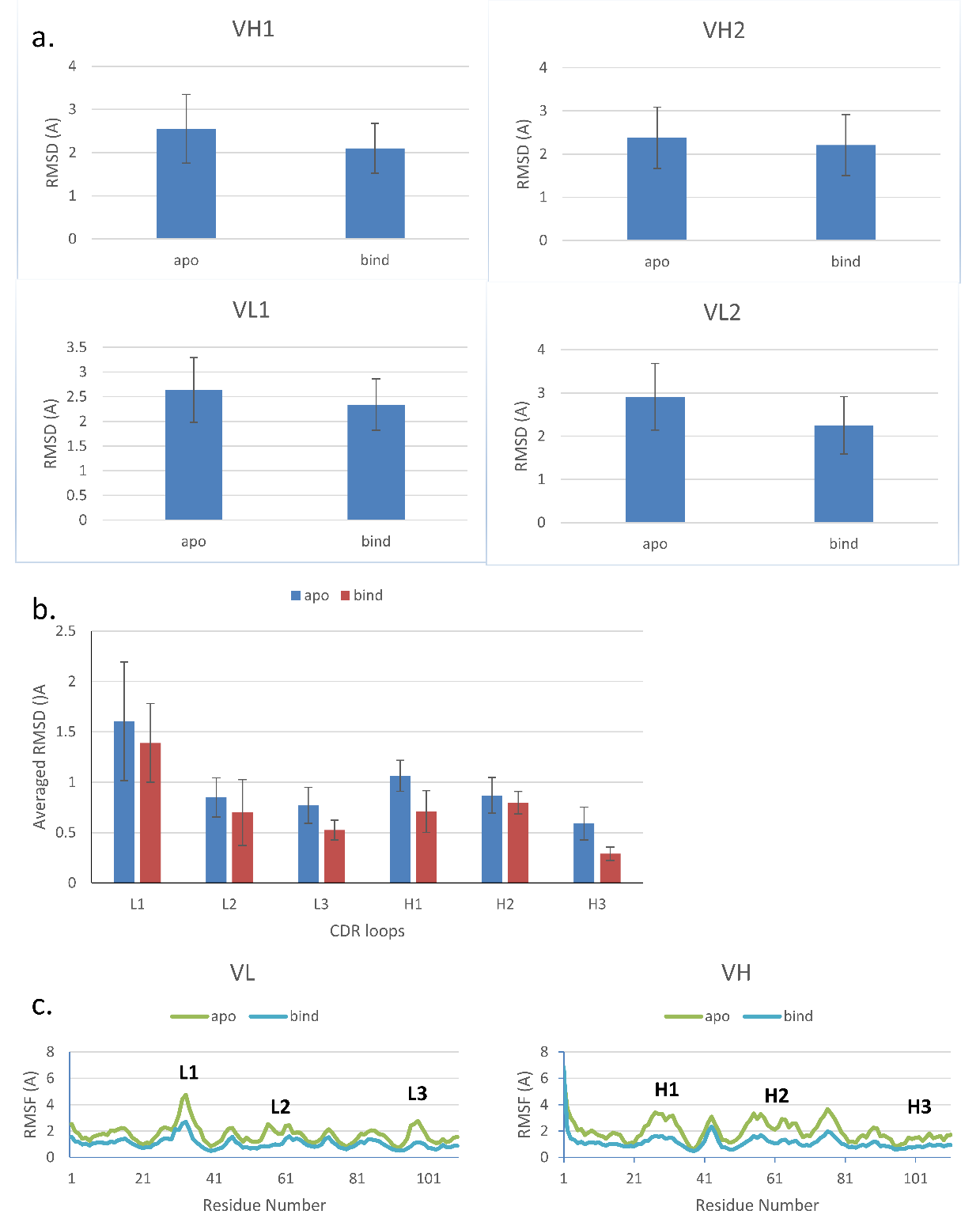


Fig. S5. Structure analysis of the paratope before and after antigen binding. a. the VH/VL orientation. b. the averaged RMSD of the six CDR loops. c. the averaged RMSF of the variable domain.

Table S1 Amino Acid sequence of the complex system.

> solanzumab light chain

DVVMTQSPLSLPVTLGQPASISCRSSQSLIYSDGNAYLHWFLQKPGQSPRLLIYKVSNRF SGVPDRFSGSGSGTDFTLKISRVEAEDVGVYYCSQSTHVPWTFGQGTKVEIKRTVAAPSV FIFPPSDEQLKSGTASVVCLLNNFYPREAKVQWKVDNALQSGNSQESVTEQDSKDSTYSL SSTLTLSKADYEKHKVYACEVTHQGLSSPVTKSFNRGEC

> solanzumab heavy chain

EVQLVESGGGLVQPGGSLRLSCAASGFTFSRYSMSWVRQAPGKGLELVAQINSVGNSTYY PDTVKGRFTISRDNAKNTLYLQMNSLRAEDTAVYYCASGDYWGQGTLVTVSSASTKGPSV FPLAPSSKSTSGGTAALGCLVKDYFPEPVTVSWNSGALTSGVHTFPAVLQSSGLYSLSSV VTVPSSSLGTQTYICNVNHKPSNTKVDKKV EPKSCDKT HTCPPCPAPELLGGPSVFLFPPKPKDTLMISRTPEVTCVVVDVSHEDPEVKFNWYVDGVEVHNAKTKPREEQYNSTYRVVSVLTVLHQDWLNGKEYKCKVSNKALPAPIEKTISKAKGQPREPQVYTLPPSRDELTKNQVSL TCLVKGFYPSDIAVEWESNGQPENNYKTTPPVLDSDGSFFLYSKLTVDKSRWQQGNVFSC SVMHEALHNHYTQKSLSLSPGK

> FcγRI

QVDTPKAVIKLQPPWVSVFQEESVTLHCEVPHLPGSSSTQWFLNGTAIQTSTPTYHITSASEDDSGEYRCQRGLSGRSDPIQLEVHRGWLLLQVSSRVLTEGEPLALRCHAWKDKLVYNVLYYRNGKAFKFFHWNSNLTILKTNMSHSGTYHCSGMGKHRYTSAGISVTVKELFPAPVLTASVTSPLLEGTPVTLSCETKLLLQRPGLQLYFSFYMGSKTLRGRDTSSEYQILTARREDSGLYWCEAATEDGNVLKRSPELELQVLGHQQPTPV

> Aβ

VHHQKLVFFAEDVGSNK

Table S2 Details about the simulation systems

| complex | conformation | Total atoms | Cations | Anion | water | Time (ns) |
| --- | --- | --- | --- | --- | --- | --- |
| solanuzumab | 1 | 567640 | 513 | 525 | 182019 | 160 |
|  | 2 | 374383 | 327 | 339 | 117724 | 40 |
|  | 3 | 473431 | 422 | 434 | 150677 | 40 |
|  | 4 | 489508 | 438 | 450 | 156025 | 40 |
|  | 5 | 424263 | 376 | 388 | 134318 | 40 |
|  | 6 | 533311 | 480 | 492 | 170598 | 40 |
|  | 7 | 408116 | 362 | 374 | 128945 | 40 |
|  | 8 | 431893 | 384 | 396 | 136856 | 40 |
|  | 9 | 448273 | 399 | 411 | 142306 | 80 |
|  | 10 | 481785 | 430 | 442 | 153456 | 40 |
|  | 11 | 481812 | 430 | 442 | 153465 | 40 |
|  | 12 | 439041 | 391 | 403 | 139234 | 40 |
| Solanuzumab+Aβ12-28 monomer | 1 | 601405 | 545 | 557 | 193070 | 160 |
|  | 2 | 374431 | 326 | 338 | 117558 | 40 |
|  | 3 | 499393 | 446 | 458 | 159132 | 40 |
|  | 4 | 533413 | 479 | 491 | 170450 | 40 |
|  | 5 | 407765 | 361 | 373 | 128646 | 40 |
|  | 6 | 627700 | 569 | 581 | 201819 | 40 |
|  | 7 | 414874 | 368 | 380 | 131011 | 40 |
|  | 8 | 480873 | 429 | 441 | 152970 | 40 |
|  | 9 | 550118 | 496 | 508 | 176007 | 80 |
|  | 10 | 473539 | 422 | 434 | 150530 | 40 |
|  | 11 | 488917 | 437 | 449 | 155646 | 40 |
|  | 12 | 498602 | 445 | 457 | 158869 | 40 |
| Solanuzumab+hFcγRI | 1 | 839657 | 766 | 779 | 271028 | 200 |
|  | 2 | 701632 | 635 | 648 | 225107 | 80 |
|  | 3 | 498942 | 441 | 454 | 157673 | 100 |
|  | 4 | 618329 | 556 | 569 | 197392 | 80 |
| Solanuzumab+hFcγRI+ Aβ12-28 monomer | 1 | 683823 | 617 | 630 | 219000 | 200 |
|  | 2 | 608696 | 543 | 556 | 194007 | 80 |
|  | 3 | 534897 | 476 | 489 | 169452 | 100 |
|  | 4 | 759611 | 690 | 703 | 244214 | 80 |
| hFcγRI | 1 | 158446 | 146 | 147 | 51223 | 120 |
